# Ultraviolet-B acclimation is supported by functionally heterogeneous phenolic peroxidases

**DOI:** 10.1038/s41598-020-73548-5

**Published:** 2020-10-01

**Authors:** Arnold Rácz, Gyula Czégény, Kristóf Csepregi, Éva Hideg

**Affiliations:** Department of Plant Biology, Faculty of Sciences, University of Pécs, Ifjúság u. 6, Pecs, 7624 Hungary

**Keywords:** Light responses, Plant stress responses, Secondary metabolism

## Abstract

Tobacco plants were grown in plant chambers for four weeks, then exposed to one of the following treatments for 4 days: (1) daily supplementary UV-B radiation corresponding to 6.9 kJ m^−2^ d^−1^ biologically effective dose (UV-B), (2) daily irrigation with 0.1 mM hydrogen peroxide, or (3) a parallel application of the two treatments (UV-B + H_2_O_2_). Neither the H_2_O_2_ nor the UV-B treatments were found to be damaging to leaf photosynthesis. Both single factor treatments increased leaf H_2_O_2_ contents but had distinct effects on various H_2_O_2_ neutralising mechanisms. Non-enzymatic H_2_O_2_ antioxidant capacities were increased by direct H_2_O_2_ treatment only, but not by UV-B. In contrast, enzymatic H_2_O_2_ neutralisation was mostly increased by UV-B, the responses showing an interesting diversity. When class-III peroxidase (POD) activity was assayed using an artificial substrate (ABTS, 2,2′-azino-bis (3-ethylbenzothiazoline-6-sulphonic acid)), both treatments appeared to have a positive effect. However, only UV-B-treated leaves showed higher POD activities when phenolic compounds naturally occurring in tobacco leaves (chlorogenic acid or quercetin) were used as substrates. These results demonstrate a substrate-dependent, functional heterogeneity in POD and further suggest that the selective activation of specific isoforms in UV-B acclimated leaves is not triggered by excess H_2_O_2_ in these leaves.

## Introduction

Hydrogen peroxide is produced in plants in a variety of metabolic and stress-inducible pathways^1^. The electron transport of chloroplasts and mitochondria as well as various peroxisomal and plasma-membrane localised oxidases produce superoxide anion radicals (O_2_^·−^), which are converted into H_2_O_2_ by the superoxide-dismutase enzyme (SOD)^2–4^. Because H_2_O_2_ is relatively stable in biological systems with a half-life of milliseconds to seconds^5^, it may act as a second messenger molecule or enzyme substrate^4–6^ in addition to being a damaging oxidising agent when present in higher concentrations^7^. Pathogens and various abiotic factors were shown to increase H_2_O_2_ production, although stress responses generally involve the activation of various H_2_O_2_ neutralising enzymes as well. Elevated H_2_O_2_ concentrations in plant leaves were documented in response to UV-B irradiation^8^, excess photosynthetically active radiation (PAR)^9^, high temperature^10–12^, drought^13^, or heavy metal stress^14^. Although H_2_O_2_ was considered capable of diffusing across membranes by itself^15^, its transport between intracellular compartments and between cells is mainly through aquaporins^16^. Such mobility facilitates the molecule’s messenger function but also requires the antioxidant control of local concentrations further away from H_2_O_2_ production sites.

Ultraviolet-exposed plants especially need to maintain an effective H_2_O_2_ regulating system^17–22^ since the UV-B (280–315 nm) component of sunlight not only elevates H_2_O_2_ concentrations *in planta* but may also photoconvert H_2_O_2_ to more hazardous hydroxyl radicals (^•^OH)^8^. Earlier studies have shown that the proper activation of class-III plant peroxidase (POD) enzymes is a key factor in the successful acclimation to UV-B both in model plants exposed to supplemental UV radiation in growth chambers^20^ and in sun leaves outdoors^23^. Our recent work with tobacco plants also showed that leaf acclimation to supplementary UV-B is realised through a selective activation of POD isoforms^22^.

There are numerous POD isoenzymes in a plant tissue, mainly in cell walls and vacuoles^24^, but phenolic peroxidases were also found in chloroplasts^25^. The common view is that POD enzymes are not substrate-selective but rather use a wide range of phenolic compounds as electron donors^26,27^ depending on the availability of these secondary metabolites^28^. At least three distinct pathways have been identified to facilitate flavonoid transport among cellular locations^29,30^, and phenolic compounds were found in a variety of cell compartments including the cytosol, vacuole, ER, as well as chloroplast and nucleus^31,32^.

Acclimative responses to UV-B include an increase in leaf phenolic contents^33,34^ and the biosynthesis of these secondary metabolites occurs under the regulation of UVR8, the UV-B photoreceptor^35^. Little is known about the molecular mechanism of UV-inducible peroxidase upregulation. UV-B-induced UVR8-regulated genes include a glutathione peroxidase in *Arabidopsis thaliana*^36^. However, to the best of our knowledge, UVR8-regulated POD genes have not been identified so far. The possibility of indirect ROS-mediated upregulation has been suggested as the mode of action of UV-B on genes encoding antioxidant enzymes^37^. Such signalling has been demonstrated to occur as an upregulating UV-B effect on the multi-function defence genes PR1 and PDFI.2^38^. This model is supported by the overlap between antioxidant responses to UV-B and several other abiotic factors^7^. Given the well-established role of H_2_O_2_ as a signal molecule, a plausible assumption is that UV-B stimulates leaf antioxidants through the increased production of ROS. In order to test this hypothesis, here, we compare the antioxidant responses of tobacco (*Nicotiana tabacum*) leaves to supplementary UV radiation and to direct H_2_O_2_ treatment. The latter was achieved as irrigation with a water solution of H_2_O_2_, which has already been shown to increase H_2_O_2_ concentrations in an experiment performed with rice seedlings^39^. The main phenolics in tobacco leaves include chlorogenic acid, rutin, and caffeic acid, but the presence of vanillic acid, ferulic acid, or quercetin has also been reported^40–45^.

The second aim of our work was to study whether UV-inducible phenolic compounds contribute to modulating leaf H_2_O_2_ concentrations as POD substrates or as direct ROS scavengers. The latter function is feasible because several phenolic compounds are highly reactive to H_2_O_2_ in vitro^46^. Regardless of their mode of action, phenolic compounds are oxidised when acting as antioxidants. This yields a wide range of products, which include phenoxyl and semiquinone radicals^47,48^. The chemistry of these reactions has been studied extensively in nutrition science^49^ and results may also be relevant to reactions assumed to occur *in planta*. However, the threat of antioxidant phenolic compounds turning pro-oxidant is less likely in plant than in animal tissues. Experiments using a specific class-III plant peroxidase, horseradish peroxidase (HRP), demonstrated that phenolic antioxidants can be regenerated from their radical forms by ascorbate (ASA)^50^, glutathione (GSH)^51^, or by monodehydroascorbate reductase^52^.

As exposure to UV-B radiation affects the way plants respond to changes in other environmental factors^53^, our experimental setup also provided an opportunity to test the following hypothesis: Do responses to exogenous H_2_O_2_ and endogenous, UV-B triggered H_2_O_2_ overlap? To this end, a two-factor treatment, H_2_O_2_ irrigation under supplemental UV-B, was also added.

## Results

Tobacco leaves were analysed from plants in four treatment groups: (1) kept under PAR only and irrigated with water (untreated control, C), (2) kept under supplementary UV-B and irrigated with water (UV-B), (3) kept under PAR only and irrigated with H_2_O_2_ solution (+H_2_O_2_), or (iv) kept under supplementary UV-B and irrigated with H_2_O_2_ solution (UV-B + H_2_O_2_). Photochemical yield measurements showed that neither UV-B nor the application of H_2_O_2_ damaged photosynthetic electron transport (Supplementary Figure S1). In fact, a slight (6–8%) increase in yield was detected in treated plants compared to untreated controls. This observation shows that leaves were not damaged by but are rather acclimated to the applied treatments.

### UV-B irradiation and H_2_O_2_ treatment induce distinct antioxidant responses

**Figure 1:**
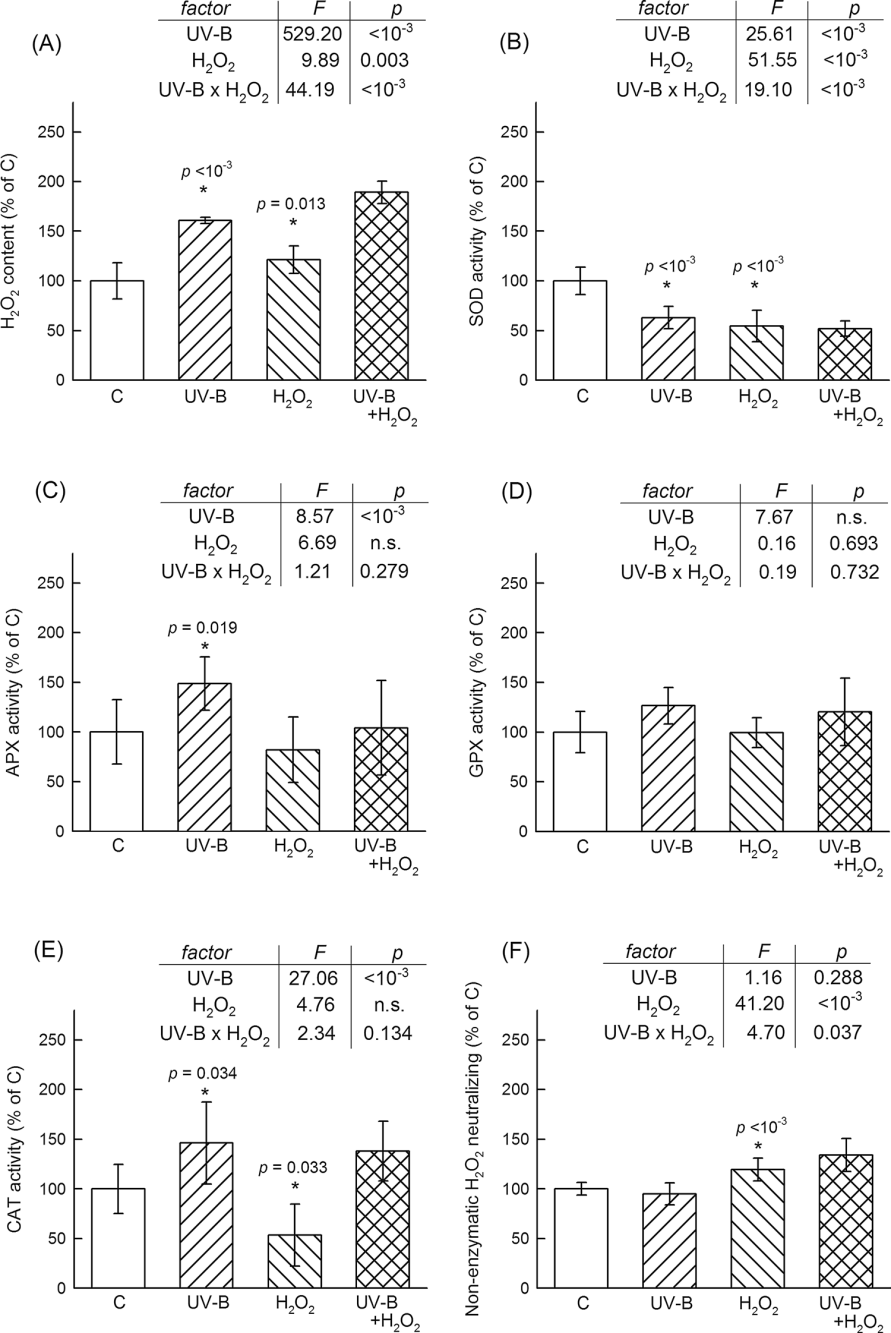
Comparison of leaf H_2_O_2_ content (**A**); activities of superoxide dismutase (**B**), ascorbate peroxidase (**C**), glutathione peroxidase (**D**), and catalase (**E**) enzymes; and non-enzymatic H_2_O_2_ neutralizing capacity (**F**) in four treatment groups: untreated control, C; UV-B treated, UV-B; H_2_O_2_ treated, H_2_O_2_; and treated with both UV-B and H_2_O_2_; UV-B + H_2_O_2_. Column heights and error bars represent means and standard deviations, respectively. 100% leaf H_2_O_2_ content = 21.49 μM mg^−1^ leaf FW, 100% SOD activity = 257.30 U mg^−1^ protein, 100% APX activity = 347.29 mU mg^−1^ protein, 100% GPX activity = 66.70 mU mg^−1^ protein, 100% CAT activity = 20.61 mU mg^−1^ protein, 100% non-enzymatic H_2_O_2_ neutralizing = 1.55 μM ASA equivalent mg^−1^ leaf FW. Columns and error bars correspond to means and standard deviations, respectively, n = 10. Significant single factor effects, such as UV-B vs. C or H_2_O_2_ vs. C, are indicated with an asterisk and *p* value above the corresponding column. Results of two-factor ANOVA are shown in insets as *F* and *p* values. Replacing a *p* value with n.s. indicates that although the corresponding *F* value was higher than *F*_*crit*_* (1,36)* = 4.11 but factor significance was not confirmed in Tukey’s post hoc test.

Leaf H_2_O_2_ contents were significantly increased by both treatments, and the effects of these were interactive: one factor had a stronger positive effect in the presence of the other (Fig. 1A). SOD activity was lower both in UV-B-exposed and in H_2_O_2_-treated plants than in untreated controls, and the interaction of the two factors was negative (Fig. 1B). APX activity was increased by ca. 50% in UV-B treated leaves but was unaffected by the H_2_O_2_ treatment either in the presence or absence of UV-B (Fig. 1C). When GPX was assayed using H_2_O_2_ as substrate, the enzyme activity showed only a non-significant marginal (*p* = 0.084) increase in UV-B treated leaves. Plant H_2_O_2_ treatment had no effect on enzyme activity (Fig. 1D). Using an organic hydroperoxide as GPX substrate revealed no differences in the activities as well (data not shown). The effects of UV-B and H_2_O_2_ on CAT activity were opposed: UV-B had a positive (ca. 47%) but H_2_O_2_ treatment had a negative (ca. − 87%) effect. Despite its negative effect as a single factor, the H_2_O_2_ treatment had no effect on how CAT activity reacted to UV-B (Fig. 1E). Non-enzymatic H_2_O_2_ neutralising capacities showed relatively small changes in response to the applied treatments. H_2_O_2_ treatment as a single factor resulted in a ca. 20% higher antioxidant capacity and UV-B alone had no significant effect. However, the two factors interacted and the H_2_O_2_ treatment resulted in a larger ca. 35% increase when UV-B was also applied (Fig. 1F).

Before examining POD responses, changes in leaf phenolics were also assessed. Dualex measurements indicated a strong (120–130%) increase in the adaxial phenolic index in response to the UV-treatment, both with and without the exogenous H_2_O_2_ treatment. The application of H_2_O_2_ alone had no significant effect (data not shown). Because the Dualex technique is based on 375 nm absorption in leaf tissue^54^, it is expected to reflect an increase in flavonoids to a larger extent than those in phenolic acid content due to differences between the UV absorption of these two compound groups^46^. TLC separation of methanolic leaf extracts was attempted to illustrate changes in phenolic compounds (Supplementary Figure S2). However, base levels in untreated leaves were too low for detection. As expected from Dualex measurements, the H_2_O_2_ treatment alone did not amend this situation. Extracts from UV-B exposed leaves, however, showed a large increase in chlorogenic acid (CGA) and the flavonol quercetin-rutinoside (RUT) contents. Since the TLC technique does not allow quantitative comparisons, our results only indicate that this marked change in phenolic composition was a common characteristic of UV-B and UV-B + H_2_O_2_ treated leaves (Supplementary Figure S2).

### UV-B responsive phenolic compounds support diverse defence functions

In order to investigate the possible contributions of the two major UV-B responsive phenolic compounds RUT and CGA to the non-enzymatic H_2_O_2_ neutralising capacity of leaves, we used pure test compounds. This is an extension of a previous study, which reported the non-enzymatic H_2_O_2_ neutralising capacities of 36 different phenolic compounds including RUT and QUE but not CGA^46^. In addition to phenolic compounds used as POD substrates in the present study, two non-phenolic antioxidants, ASA and GSH, were also added due to their potential to re-reduce oxidised phenolics^50,51^. The results are shown in Table 1, relative to the non-enzymatic H_2_O_2_ neutralising capacity of ASA. The two phenolic acids, CGA and caffeic acid (CAA), were weaker antioxidants than ASA and much weaker than the two flavonols; their H_2_O_2_ neutralising capacities were similar to those of GSH in this in vitro model. Following this comparison of direct H_2_O_2_ reactivities, we compared phenolic compounds as POD substrates.

**Table 1:** Non-enzymatic H_2_O_2_ neutralizing capacities.

Compound	H_2_O_2_ neutralizing capacity (µM compound in µM ascorbic acid equiv.)
Caffeic acid (CAA)	0.55
Chlorogenic acid (CGA)	0.69
Glutathione (GSH)	0.57
Quercetin (QUE)	3.50
Quercetin-rutinoside (RUT)	1.65
Ascorbic acid (ASA)	1.00

**Figure 2:**
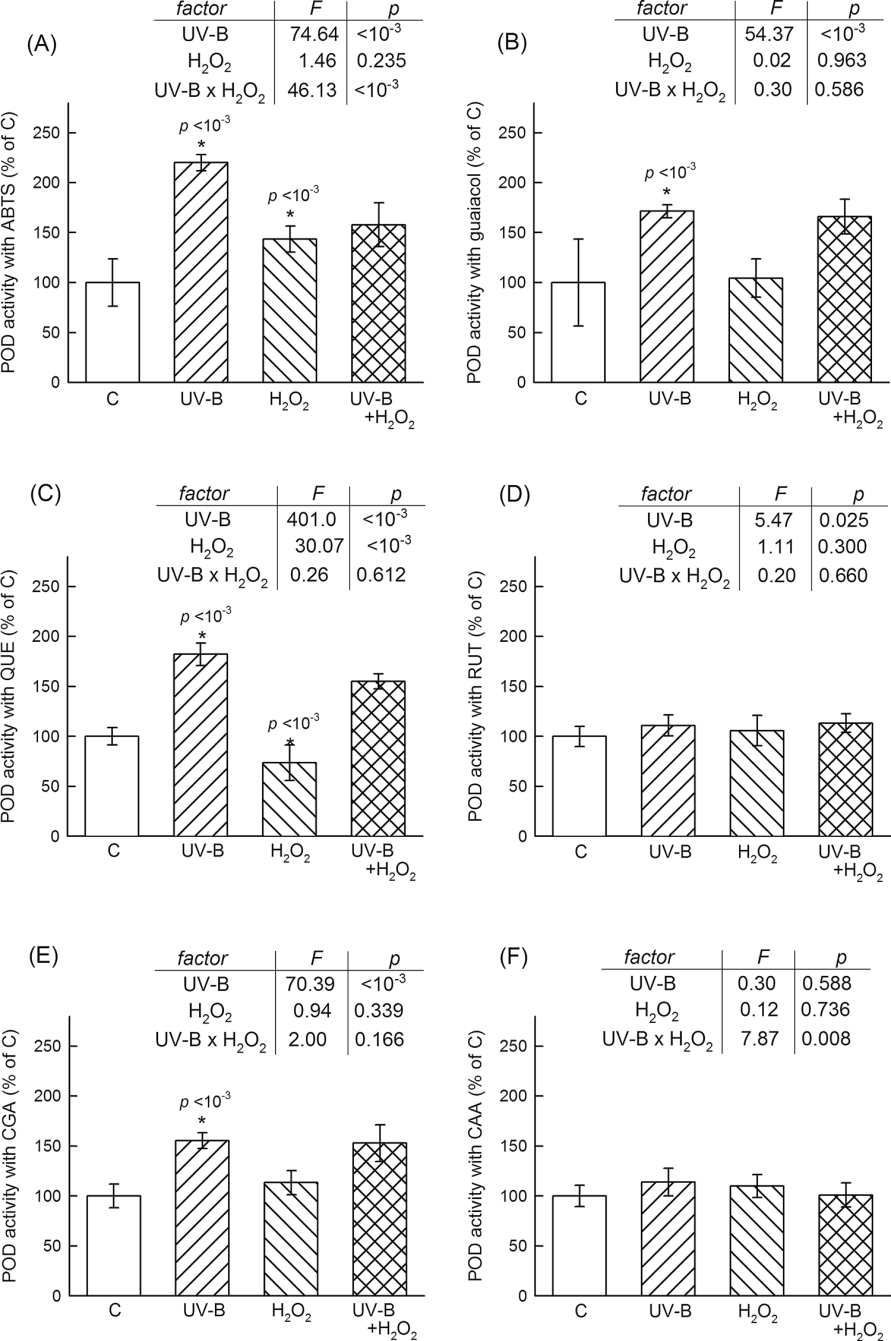
Comparison of class-II peroxidase enzyme activities assayed with various substrates, ABTS (**A**), guaiacol (**B**), quercetin (**C**), quercetin-rutinoside (**D**), chlorogenic acid (**E**) or caffeic acid (**F**) in four treatment groups: untreated control, C; UV-B treated, UV-B; H_2_O_2_ treated, H_2_O_2_; and treated with both UV-B and H_2_O_2_; UV-B + H_2_O_2_. Column heights and error bars represent means and standard deviations, respectively. All values are shown as percentage of the mean of values measured in untreated plants. 100% POD activities as U mg^−1^ protein were the following: 5.97 with ABTS, 1.77 with guaiacol (Gua), 0.93 with quercetin (QUE), 88.46 with quercetin-3-*O*-rutinoside (RUT), 43.51 with chlorogenic acid (CGA), and 2.66 with caffeic acid (CAA). Columns and error bars correspond to means and standard deviations, respectively, n = 10. Significant single factor effects, such as UV-B vs. C or H_2_O_2_ vs. C, are indicated with an asterisk and *p* value above the corresponding column. Results of two-factor ANOVA are shown in insets as *F* and *p* values.

Using ABTS or guaiacol as substrates is a common practice when POD activities are assayed. We complemented these two methods by using four different phenolic compounds that occur in tobacco leaves. CGA and RUT were chosen because these were present in higher amounts in UV-B treated leaves (Supplementary Figure S2). The choice of the aglycone form of RUT (quercetin, QUE) was based on the earlier use of this substrate to assess POD in tobacco leaves^22^. CAA was included in the study as a reported major phenolic component of tobacco leaves^55^. In the following, POD activities are discussed according to the substrate used in the assay; for example, RUT-POD refers to POD activity measured using RUT as substrate. Figure 2 shows that the results were strongly affected by the choice of substrate, both in activity (enzyme units) and in terms of responses to treatments. POD enzyme units measured in untreated leaves using various substrates followed a RUT-POD > CGA-POD > ABTS-POD > CAA-POD > Gua-POD > QUE-POD > order. Activities measured in untreated leaves with these substrates are given in legends to Figs.1 and 2 in enzyme units. CAA-POD and RUT-POD showed no change in response to either treatment (Fig. 2D,F, respectively). ABTS-POD increased in response to UV-B and H_2_O_2_ treatment by ca. 120% and 30%, respectively, compared to untreated leaves. The positive effect of UV-B was maintained in H_2_O_2_ treated plants, although the two factors interacted and the H_2_O_2_ treatment lessened the extent of the positive UV-B effect (Fig. 2A). Gua-POD and CGA-POD was increased by UV-B regardless of the application of H_2_O_2_ treatment, which was not a significant factor. Interestingly, the two treatments had opposite effects on QUE-POD: the UV-B treatment increased the enzyme activity, while the H_2_O_2_ treatment decreased it. These effects were maintained as significant in the two factor treatment without interaction, and the positive effect of UV-B was smaller without the H_2_O_2_ treatment (Fig. 2C).

**Figure 3:**
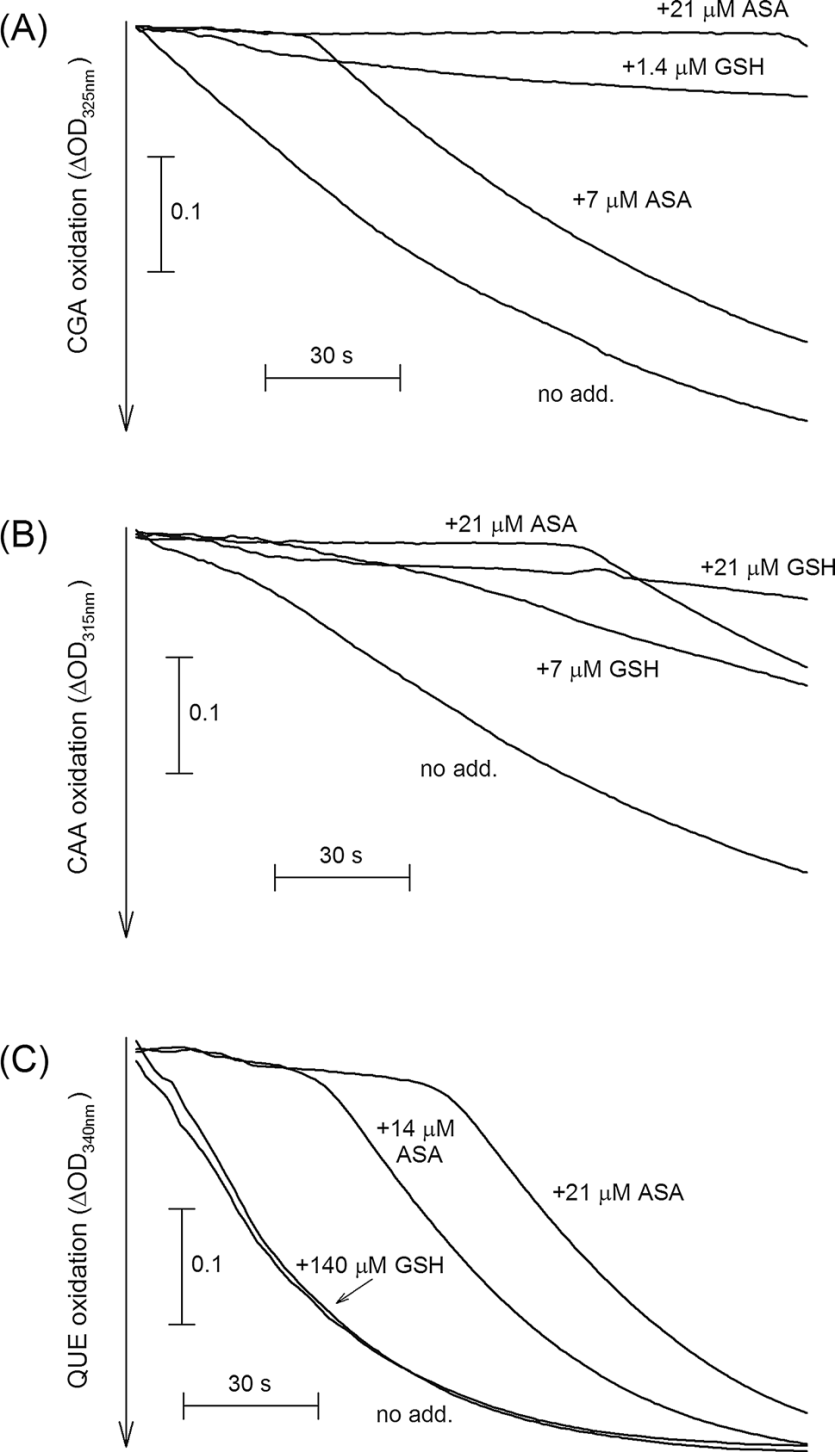
The effect of ascorbate (ASA) or glutathione (GSH) on the oxidation of phenolic compounds (**A**) chlorogenic acid, CGA, (**B**) caffeic acid, CAA, and (**C**) quercetin, QUE as POD substrates. All substrates were used at 3 mM concentrations. Using molar extinction coefficients from Table 2, ΔOD = 0.1 corresponds to the oxidation of 83 µM CGA (**A**), 62 µM CAA (**B**) or 387 µM QUE (**C**).

In the following section, we studied the effects of ASA or GSH on the oxidation rates of QUE, RUT, CAA, or CGA as POD substrates. In this experiment, various amounts of ASA or GSH were added and the kinetics of phenolic substrate oxidation by POD enzymes contained in the leaf extract was followed photometrically at the indicated wavelengths. ASA and GSH were used in the 1.4 to 140 μM concentration range and the ability of the reactivity of these antioxidants to restore oxidised phenolic substrates was illustrated by a time delay in the consumption of these compounds. In this experiment, we used a pooled sample of UV-B treated leaves because these had the highest relative POD activities (Fig. 2). The oxidation rate of RUT was not affected by the presence of either ASA or GSH up to 140 μM concentrations (data not shown). Figure 3 shows that GSH was most reactive to oxidised CGA (Fig. 3A), less reactive to oxidised CAA (Fig. 3B), and did not restore oxidised QUE, even at the highest applied concentration (Fig. 3C). The efficiency of the same concentration (20 μM) ASA to retard phenolic substrate oxidation followed an opposite QUE > CAA > CGA order (Fig. 3).

## Discussion

Using model plants in growth chambers, the present work shows that UV-B irradiation selectively enhances a subset of the antioxidant network. Analysing data from publications, which reported UV-induced changes in antioxidant enzyme activities, we have already shown that the stronger activation of peroxidases than SOD is a special characteristic of acclimative plant responses to UV-B^20^. The role of this response is to avoid high leaf H_2_O_2_ levels prone to UV-B photocleavage into hydroxyl radicals^8^. The present study supports this model. Moreover, it shows that successful acclimation (avoided loss in leaf photochemical yield) may also be realised with a decrease in SOD activity combined with increased APX, POD, and CAT activities (Figs. 1 and 2). In the present experiment, GPX activities were not affected by the applied UV-B treatment (Fig. 1D), contrary to earlier reports on UV-B regulated GPX expression in Arabidopsis^56^. This may be due to differences in the UV-B fluence rates applied and in the levels of detection (gene vs. enzyme activity). CAT activities were significantly lower (20.6 and 30.1 mU mg protein^−1^ control and in UV-B acclimated leaves, respectively) than those of APX (347 and 516 mU mg protein^−1^) or POD (1–100 U mg protein^−1^, depending on substrate and treatment), which is probably due to the low photorespiration in leaves grown under relatively low PAR in this experiment. POD responses to UV-B showed an interesting heterogeneity and suggest that only specific isoforms contribute to the acclimation. Our earlier study has already demonstrated that two POD assays using traditional substrates, ABTS and guaiacol, registered different extents of POD activation in UV-B-treated tobacco leaves^22^. Using four phenolic compounds naturally occurring in tobacco leaves (CAA, CGA, RUT, QUE), we show here that although these can be oxidised as POD substrates, only specific POD isoforms contribute to acclimation to supplemental UV-B (Fig. 2). This conclusion is similar, but not identical, to that of Jansen et al.^57^. Studying the UV-susceptibility of transgenic tobacco lines over-expressing phenol-oxidising peroxidases, the authors put forward a model in which isoenzyme diversity resulted in the polymerisation and/or crosslinking of specific phenolic compounds, and which increased the protection of plants from UV-radiation^57^. While not debating the validity of their model, we offer an alternative, which can be coexisting in leaves. Our hypothesis is based on the assumptions that (1) phenolic compounds protect leaves from radiation not only as UV screening compounds but also as antioxidants, (2) phenolics oxidatively modified by either POD or in a direct reaction with H_2_O_2_ can be re-generated, and (3) the assignment of individual compounds to defence functions depends not only on their antioxidant capacities but also on the metabolic economy of their regeneration.

Several authors have already shown that the phenolic compounds included in our study act as electron donors to a specific POD form: HRP^58,59^). Our data show that these are also capable of supporting other POD, such as the ones in tobacco leaves, although the biochemical properties of the tobacco enzyme were found to be distinct from those of HRP^60^. First, we discuss the possible roles of the two phenolic components abundant in UV acclimated leaves, CGA and RUT. Even in the absence of the UV treatment, both compounds were efficient POD substrates, conferring 15 to 90-times higher POD activities than CAA or QUE in untreated leaves. The complexity of the phenolic defence response is supported by the result that substrates favoured by UV-responsive POD isoforms (CGA and QUE, Fig. 2) do not fully correspond to compounds accumulated in high amounts in UV-treated leaves (CGA and RUT, Supplementary Fig. 2). However, while CGA-POD was ca. 50% more active in UV-B exposed leaves than in controls, there was no significant change in RUT-POD (Fig. 2E,D, respectively). On the other hand, RUT is a strong direct H_2_O_2_ neutralising antioxidant, with 1.65-times higher reactivity to ROS than ASA (Table 1). Therefore, the explanation for the substantial increase in leaf RUT content in UV-exposed leaves is not the increased need for this compound as electron donor to POD but rather as a direct antioxidant. Contrary to RUT, CGA is a relatively poor non-enzymatic antioxidant (Table 1) but an efficient POD substrate. POD-oxidised CGA was efficiently recovered by ASA or GSH. The former finding is in agreement with earlier reports using HRP^58,61^, and the latter is a novel one. We found no such recovery in the case of POD-oxidised RUT. This difference also supports the participation of CGA-POD, but not RUT-POD, in the observed UV response. CGA biosynthesis is reportedly induced by a variety of stress conditions in addition to UV^62^, and our results suggest that its main role is lessening damage as a POD substrate.

Considering the other two phenolic leaf components, CAA and QUE, the amounts of these were either unaffected by the UV exposure or the increase was minor and below the detection threshold of the applied TLC method. CAA is a relatively weak direct antioxidant (Table 1) and the relatively low POD activity it conferred (6% of CGA-POD) did not change upon UV exposure (Fig. 2F). These observations suggest that the role of CAA in the UV response is negligible. QUE, an aglycone flavonol, is of more interest. First, flavonoid aglycones are usually present in leaves in much lower amounts than their glycosylated forms, such as RUT^63^. Thus, the low activity of QUE-POD (ca. 90-times lower than RUT-POD and 45-times lower than CGA-POD in untreated leaves) may be explained by a relatively low amount of POD isoforms preferring a substrate in short supply. However, the significance of QUE in the UV response should not be dismissed. QUE is a very efficient direct H_2_O_2_ antioxidant, 3.5-times stronger than ASA. Also, the relative activity of QUE-POD may be low, but it nearly doubled in response to UV-B. On the other hand, the regeneration of QUE from its oxidised form is the least ‘economical’ among the phenolic compounds in this study in the sense that it required more ASA than the restoration of CGA or CAA, and it was not recovered by GSH (Fig. 3). This result also suggests that the QUE form yielded by leaf QUE-POD is a phenoxyl radical rather than a GSH-reactive semiquinone, similar to the form identified in animal cells^51^.

In summary, the antioxidant aspect of tobacco leaf UV acclimation was realised mainly through lowered SOD and increased peroxidase activities; the latter involving isoforms that use CGA and, to a smaller extent, QUE as electron donors. Although the increased RUT pool indicates the potential of more efficient non-enzymatic H_2_O_2_ neutralisation, there was no significant change in this function in UV exposed leaves (Fig. 1F), indicating that the contribution of RUT to this pathway was minor.

Nevertheless, H_2_O_2_ levels were higher in these UV-B-acclimated leaves than in controls (Fig. 1A). A UV-inducible increase in leaf H_2_O_2_ concentrations has already been reported in stressed plants, where the irradiation resulted in a decrease in photosynthetic performance^8^ but not yet in well-acclimated ones. Hydrogen peroxide is a well-established secondary messenger^1^ and it is plausible that controlled low levels of this ROS participate in the induction of the antioxidant response to UV-B as well. Whether acclimative UV-responses are triggered by UV-B directly, through the UVR8 photoreceptor^35^ or by UV-B induced elevated H_2_O_2_ levels, is still an open question. The former model would result in a large number of specifically UV-B responsive peroxidase genes, but so far, the only example is the GPX encoding Arabidopsis AT4G35870^35,36,56,64^. In this study, we compared antioxidant responses to UV-B and exogenous H_2_O_2_ at functional (activity) levels. The manner in which irrigation with H_2_O_2_ increases the leaf concentrations of this ROS is unknown so far, but cross membrane transport^16^ and NADPH oxidase activation^7^ are among possible candidates. Wan and Liu^39^ found that 0.6–15 mM H_2_O_2_ root application resulted in a 50–200% increase in rice leaf H_2_O_2_ content and caused oxidative membrane damage, a strong decline in photosynthesis and APX down-regulation. In our experiment, a lower concentration (0.1 mM) was used for soil irrigation, and the treatment resulted in a 20% increase in leaf H_2_O_2_ levels (Fig. 1) but did not cause any loss in leaf photochemical yield. APX activity was unaffected, and the only positive effect on POD was detectable as ABTS-POD. None of the phenolic-substrate-using POD isoforms were stimulated by the H_2_O_2_ (Fig. 2), confirming that the synthetic compound ABTS as electron donor assesses a different subset of leaf POD than natural compounds. Contrary to the UV-B treatment, which enhanced the enzymatic but not the non-enzymatic neutralisation of H_2_O_2_, the direct ROS treatment applied increased the latter defence pathway but had only a minor positive effect on enzymatic defence. Moreover, QUE-POD and CAT activities were lower (by 25% and 50%, respectively) in H_2_O_2_ treated leaves than in controls. The only common response to the two different treatments was a decrease in SOD activity. This suggests that the source of neither UV-B-induced nor H_2_O_2_-irrigation-induced excess H_2_O_2_ is an increased enzymatic conversion of superoxide radicals. Because exogenous H_2_O_2_ resulted in an increase in non-enzymatic antioxidant capacity without a marked increase in phenolic content, the contribution of these compounds as direct H_2_O_2_ scavengers was most likely minor in acclimating to this treatment.

Factor interactions between UV-B and H_2_O_2_ treatments were explored further using two-way ANOVA. Statistically verified interactions, which do not grant but only imply the possibility of crosstalk between the two factors, require two conditions: One is that single factors are significant in the two-factor experiment, for example, UV-B increasing the studied effect (e.g. enzyme activity) both in the absence and in the presence of exogenous H_2_O_2_ (*p* < 0.05 in the first row of the inset tables in Figs. 1 and 2) and vice versa (*p* < 0.05 in the second row). The second condition is a *p* < 0.05 interaction (the third row in these tables). These two conditions are met only in the case of leaf H_2_O_2_ content (Fig. 1A) and SOD (Fig. 1B). The effect of treatments was positive in the former and negative in the latter case. Lowering SOD-mediated production is possibly a common acclimative response to an increase in cellular H_2_O_2_ levels, regardless of the nature of the external stimulus. A parallel application of UV-B and H_2_O_2_ resulted in an additive effect on H_2_O_2_ content in the sense that the simultaneous presence of the two factors led to an effect that was equal to the sum of the effects caused by the two factors applied separately^53^.

The major differences in antioxidant responses to H_2_O_2_ and UV-B, as well as the very limited interaction between the two factors when applied in parallel, as discussed above, suggest that UV acclimation is unlikely to have been brought about by the UV-induced increase in leaf H_2_O_2_ content. A difference in production H_2_O_2_ sites in response to the two treatments may argue against this assumption, but the relatively long life-time and ability of this ROS to spread in tissues^16^ diminishes the importance of this aspect. In the absence of evidence for UVR8-initiated activation of acclimative antioxidant signalling, one can only speculate on potential routes. If this pathway involves direct UV perception, then candidates include a UV-B photoreceptor distinct from UVR8^65^ or a contribution of UV-A photoreceptors^64^ as the broad-band UV source applied in our experiment contained UV-A as well. A metabolite initiated pathway may include oxidised ascorbate, which has already been implicated in responses to stressors other than UV radiation^66^ or possibly, oxidised phenolic compounds.

Increased phenolic peroxidase activity has been widely reported as a general, non-specific defence response. Our present study, however, shows the existence of inducement-specific, phenolic substrate-dependent POD responses in UV-treated leaves and suggests a further investigation of the heterogeneity of POD responses under different abiotic stress conditions. Further, the present study also draws attention to the possibility of the novel yet unexplored complexity of POD responses to other stress conditions as well. As illustrated by the example of the UV-induced changes, an increase in a certain phenolic component in the leaf does not necessarily correspond to its increased use as POD substrate; thus, the latter cannot be fully explained by its increased availability. A more plausible model is the selective upregulation of POD isoforms using phenolic substrates, which can be recovered from their oxidised form by relatively low amounts of other antioxidants, such as ascorbate or glutathione. This hypothesis is supported by our data but must be verified further through a quantitative analysis of antioxidant metabolites and correlations between changes in their levels during UV acclimation.

## Methods

### Chemicals, plant material, and treatments

Pure phenolic compounds quercetin, rutin, caffeic acid, and chlorogenic acid were purchased from Extrasynthese S.A.S. (Genay, Rhone, France). Hydrogen peroxide was obtained from VWR International (Debrecen, Hungary). Other chemicals were purchased from Sigma-Aldrich Kft (Budapest, Hungary). Tobacco (*Nicotiana tabacum* cv. Xanthi) plants were grown in growth chambers (Fitotron, SGC 120 Plant Growth Chamber, Weiss Technik UK, Loughborough, UK) under 150 μmol m^−2^ s^−1^ photosynthetically active radiation (PAR) using long day conditions (16/8 h, 25/20 °C). Four weeks after emergence, the plants were divided into four groups, each containing 4 plants: (1) treated with exogenous H_2_O_2_, (2) exposed to supplemental UV radiation, (3) H_2_O_2_ + UV, and (4) untreated controls. Plants in the two H_2_O_2_ treatment groups were irrigated with 100 mL 100 μM H_2_O_2_ daily and plants in the two other groups received equal volumes of water. UV radiation was provided by Q-Panel UVB-313EL tubes (Q-Lab Ltd., Bolton, UK) wrapped in a single layer of cellulose diacetate filter (Courtaulds Chemicals, Derby, UK), and it was measured in the growth chamber with a spectroradiometer (Flame, Ocean Optics, Largo FL, USA). The spectrum was centred at 311 nm (Supplementary Figure S3), and irradiation for 4 h (between 10 a.m. and 2 p.m. daily) provided 6.9 kJ m^−2^ d^−1^ biologically effective UV-B (280–315 nm) calculated using the biological spectral weighting function developed for plants^67^. At the end of the treatment period, non-invasive leaf measurements were performed; thereafter, detached leaves were frozen in liquid N_2_ and stored at − 80 °C until used for either chromatography analysis or antioxidant capacity assays. Each measurement was carried out using the fully expanded leaf of the 4th node, in order to exclude effects of age-related heterogeneity of the UV response^22^.

### Chlorophyll florescence measurements

Plants were kept in darkness for 30 min before photochemical yields and non-photochemical quenching were characterised by chlorophyll fluorescence-derived parameters using the MAXI-version of the Imaging PAM (Heinz Walz GmbH, Effeltrich, Germany). At the end of the dark adaptation period, a saturating pulse was applied in order to measure the minimum and maximum fluorescence yields (F_0_: before the pulse and F_m_: after the pulse). Following this, the leaf was illuminated with blue actinic light corresponding to 110 μmol m^−2^ s^−1^ PAR applied for 4 min, then the F′ and F′_m_ fluorescence yields were measured before and after a saturating pulse. The maximum and effective Photosystem (PS) II quantum yields were calculated from these data as F_v_/F_m_ = (F_m_ − F_0_)/F_m_. and Y(II) = (F′_m_ − F′)/F′_m_, respectively^68^. Non-regulated and regulated non-photochemical energy dissipation processes were characterised by Y (NO) = F′/F_m_ and Y(NPQ) = F′/F′_m_ − F′/F_m_, respectively^69^.

### Assessments of leaf phenolic contents

Leaf flavonoid content was estimated using a non-invasive optical method on both adaxial and abaxial sides with a Dualex Scientific optical sensor (ForceA, Orsay, France). This method is based on the absorption in leaf tissue at 375 nm^54^. In addition, phenolic compounds were analytically separated with thin-layer chromatography (TLC) performed on silica gel-coated aluminium sheets (60 F_254_, 12 × 20 cm; Merck KGaA, Darmstadt, Germany). Frozen leaves were powdered in liquid N_2_ using a pestle and mortar and extracted in 70% methanol. Leaf extracts (0.3 mg leaf FW mL^−1^) and test compounds (1 mg mL^−1^) were applied on the TLC plate at 5 μL volumes. The developing buffer was a 30 mL mixture of ethyl acetate, formic acid, acetic acid, and water (100:11:11:27, V:V:V:V). The plate was first air-dried, then dried in a desiccator for 20 min. Phenolic compounds were detected under UV light centred at 365 nm (VL215.L, Vilbert Lormat, France) after spraying the plate with a NaturStoff solution^70^.

### Enzyme activity measurements

Frozen leaves were powdered in liquid N_2_ using a pestle and mortar and extracted in an ice cold sodium-phosphate buffer (50 mM, pH 7.0) containing 1 mM EDTA. Leaf homogenates were centrifuged (24.400×*g* for 30 min at 4 °C, Hettich Rotina 380 R, Andreas Hettich GmbH, Tuttlingen, Germany) and supernatants were kept at − 20 °C until use. Protein contents were determined using the standard Bradford assay^71^.

POD (EC 1.11.1.7) activities were measured in acidic reaction mixtures (50 mM phosphate citrate buffer, pH 5.0) using six different substrates. The reactions also contained 400 μM H_2_O_2_ in all cases, and one of the following substrates: (1) ABTS (2,2′-azino-bis(3-ethylbenzothiazoline-6-sulphonic acid)), (2) guaiacol (2-methoxyphenol), (3) caffeic acid ((2E)-3-(3,4-Dihydroxyphenyl)prop-2-enoic acid), (4) chlorogenic acid ((1S,3R,4R,5R)-3-{[(2E)-3-(3,4-dihydroxyphenyl)prop-2-enoyl]oxy}-1,4,5-trihydroxycyclohexanecarboxylic acid), (5) quercetin (2-(3,4-dihydroxyphenyl)-3,5,7-trihydroxy-4H-1-benzopyran-4-one) + 14 mM ascorbate, or (6) rutin (2-(3,4-dihydroxyphenyl)-5,7-dihydroxy-3-[α-L-rhamnopyranosyl-(1 → 6)-β-d-glucopyranosyloxy]-4H-chromen-4-one). Enzyme activities were quantified following the oxidation of the corresponding substrate as absorption change using a spectrophotometer (Shimadzu UV1800, Shimadzu Corp., Kyoto, Japan). Substrate concentrations in the reaction mixture and absorbance wavelengths are summarised in Table 2 along with the molar extinction coefficients used to calculate enzyme activities as mU activity mg^−1^ protein, where 1 U = 1 mM substrate min^−1^. References describing the details of original methods are also listed in Table 2. When indicated, the reaction mixture contained either ascorbate (7–14 µM) or GSH (1.4–140 µM) in addition to one of the phenolic compounds.

**Table 2:** Characterization of POD enzyme substrates used in the present study.

Substrate	Concentration (mM)	Absorbance wavelengths (nm)	Molar extinction coefficient (ε, mM^−1^ cm^−1^)	Reference
ABTS	183	765	11.23	^72^
GUA	2	450	5.98	^73^
CAA	3	315	0.62	This study
CGA	3	325	0.83	This study
QUE	3	340	3.87	*^58^
RUT	3	265	0.03	This study

*Due to the fast oxidation of quercetin a substrate, the reaction was followed by the loss of ascorbate due to recovering oxidized quercetin at 295 nm, using ε_295 nm_ = 1.47 mM^−1^ cm^−1^.

Superoxide dismutase (SOD, EC 1.15.1.1) activity measurements were carried out according to Sun et al.^74^, based on the inhibition of 0.1 mM nitroblue tetrazolium (NBT) reduction by xanthine–xanthine-oxidase (2 mM and 25 mU, respectively), which generated superoxide anions, and activity was determined as U SOD mg^−1^ protein.

Ascorbate peroxidase (APX, EC 1.11.1.11) activity was measured according to Nakano and Asada^75^ by following the oxidation of ascorbate at 295 nm in a sodium phosphate buffer (50 mM, pH 7.0) containing 1 mM EDTA, 0.5 mM ascorbate, and 1 mM H_2_O_2_. The results were corrected for APX-independent H_2_O_2_ reduction, which was typically less than 10% of enzymatic rates. Enzyme activities were calculated using the molar extinction coefficient of ascorbate (ε_295 nm_ = 1.47 mM^−1^ cm^−1^) as mU APX mg^−1^ protein.

Glutathione peroxidase (GPX, EC 1.11.1.9) activity was measured by following the NADPH oxidation at 340 nm according to Lawrence and Burk^76^. The reaction mixture contained 1 mM EDTA, 0.2 mM NADPH, 1 mM NaN_3_, 1 mM reduced glutathione, and 1 U mL^−1^ glutathione reductase in 50 mM potassium phosphate buffer (pH 7.0) and either 0.25 mM H_2_O_2_ or 0.25 mM cumene hydroperoxide. NADPH oxidation was followed at 340 nm (ε = 6.42 mM^−1^ cm^−1^) and enzyme activities were determined as mU GPX mg^−1^ protein.

Catalase (CAT, EC 1.11.1.6) activity was determined as described by Aebi et al.^77^ by following the decrease in H_2_O_2_ concentration as 240 nm absorbance in a reaction mixture containing 18.6 mM H_2_O_2_ and 1 mM EDTA in a 50 mM sodium-phosphate buffer (pH 7.0). The reaction was started by adding 60 µL leaf sample (corresponding to 1.3–4.1 µg soluble protein) and CAT activities were given as mU mg^−1^ protein.

### Non-enzymatic H_2_O_2_ antioxidant capacity measurement

Hydrogen peroxide neutralising antioxidant capacities were evaluated through the photometric detection of iodine (I_2_) yielded in the reaction between H_2_O_2_ and potassium iodide (KI), and the ability of H_2_O_2_ reactive compounds to lessen the amount of this product^78^. For this experiment frozen leaves were powdered in liquid N_2_ using a pestle and mortar and extracted in 70% (v/v) ethanol. The reaction mixture contained 25 μM H_2_O_2_, 595 μM KI in potassium-phosphate buffer (pH 7.0) and either leaf extracts (corresponding to 300 μg leaf FW) or one of the pure test compounds (0.42–3.2 mM). The final concentration of ethanol in the reaction mixture was always 7.5% (v/v). Absorption at 405 nm was measured twice, immediately and 3 min after mixing assay components using a Multiskan FC plate reader (Thermo Fischer Scientific, Shanghai, China). Non-enzymatic H_2_O_2_ antioxidant capacities were given as μM ascorbic acid (ASA) equivalents.

### Hydrogen peroxide content measurement

Leaf H_2_O_2_ levels were estimated using a photometric assay^79^ based on the H_2_O_2_-induced absorption change of 125 μM xylenol orange in 6% (v/v) trichloroacetic acid (TCA). For this assay, samples were collected from plants within the growth chamber under light conditions corresponding to treatment groups i.e. PAR only or PAR plus UV-B. Three leaf disks corresponding to 26–56 mg FW were homogenised in 6% TCA immediately after cutting, centrifuged (15,000×*g*, 10 min, 4 °C, Heraeus Fresco 17 Centrifuge, Thermo Fisher Scientific, Waltham, USA), and the supernatants were incubated for 30 min before detecting 560 nm absorptions. Leaf H_2_O_2_ contents were given in nM mg^−1^ FW units using calibration curves in the 0–10 nM H_2_O_2_ range.

### Statistical analysis

Each treatment group contained four plants. One leaf from each plant was chosen for the analyses, and all measurements were performed in 3–4 repetitions. Results are presented as means ± standard deviations. The combined and single factor effects of UV-B and H_2_O_2_ were analysed with a two-way ANOVA. Three null hypotheses were tested: (1) the H_2_O_2_ treatment had no effect, (2) the absence/presence of UV-B over the PAR background had no effect, and (3) there was no interaction between the two factors. Tukey HSD was used as post-hoc test and verified rejections of the ANOVA null hypotheses were characterised with *p* values. Statistical analyses were performed using the PAST software^80^.

## Supplementary information

Supplementary information

## Abbreviations

ABTS: 2,2′-Azino-bis (3-ethylbenzothiazoline-6-sulphonic acid)
ABTS-POD: Peroxidase activity measured with ABTS as substrate
APX: Ascorbate peroxidase, EC 1.11.1.11
CAA: Caffeic acid
CAT: Catalase, EC 1.11.1.6
CGA: Chlorogenic acid
Fv/Fm: Maximum quantum efficiency of PS II
GPX: Glutathione peroxidase, EC 1.11.1.9
guaiacol: 2-Methoxyphenol
Gua-POD: Peroxidase activity measured with guaiacol as substrate
HRP: Horseradish peroxidase
PAR: Photosynthetically active radiation
QUE: Quercetin
QUE-POD: Peroxidase activity measured with QUE as substrate
POD: Peroxidase, EC 1.11.1.7
RUT: Quercetin-3-*O*-rutinoside, rutin
RUT-POD: Peroxidase activity measured with RUT as substrate
SOD: Superoxide dismutase, EC 1.15.1.1
TLC: Thin-layer chromatography
UV-B: 280–315 nm ultraviolet radiation
Y(II): Quantum efficiency of light acclimated PS II
Y(NO): Non-regulated non-photochemical quenching of PS II
Y(NPQ): Regulated non-photochemical quenching of PS II
