# Supplementary material for: Ultraviolet-B acclimation is supported by functionally heterogeneous phenolic peroxidases
Source: Sci Rep. 2020 Oct 1;10:16303. doi: 10.1038/s41598-020-73548-5 (PMC7530754; doi:10.1038/s41598-020-73548-5)
Supplement: Supplementary file 1 — Supplementary information [file 41598_2020_73548_MOESM1_ESM.pdf]

Supplementary material

Ultraviolet-B acclimation is supported by functionally heterogeneous phenolic peroxidases

Arnold Rácz, Gyula Czégény, Kristóf Csepregi, Éva Hideg

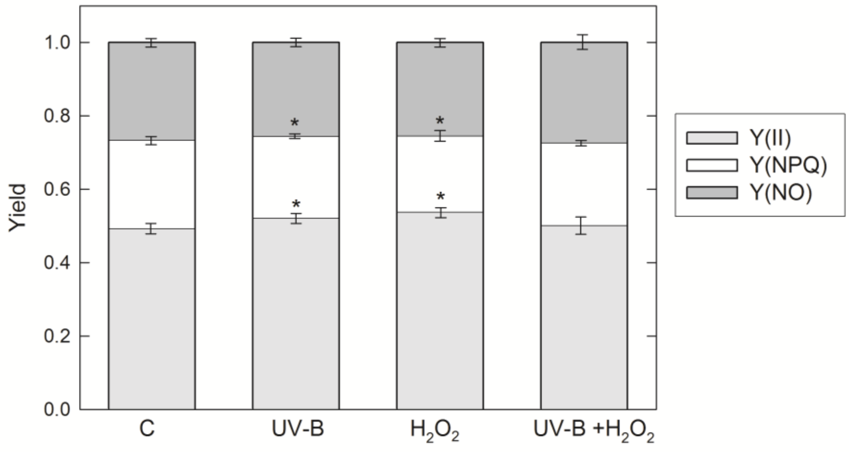

Supplementary Figure 1.

Changes in leaf photochemical yield Y(II), regulated non-photochemical quenching Y(NPQ) and non regulated non-photochemical quenching Y(NO) in four treatment groups: untreated (C), H_2O_2 treated (H_2O_2), UV-B treated (UV-B), or treated with both UV-B and H_2O_2 (UV-B + H_2O_2).

Column heights and error bars represent means and standard deviations, respectively, n=4. Significant single factor effects, such as difference between UV-B and C or H_2O_2 and C leaves, are indicated with an asterisk. Two-factor ANOVA analysis did not identify significant factorial effects or interaction (data not shown).

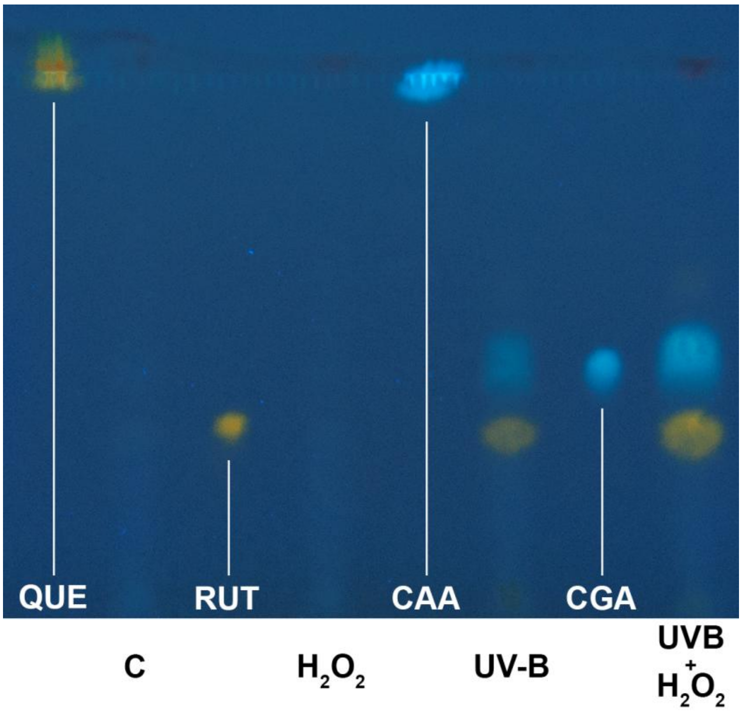

Supplementary Figure 2.

Thin layer chromatography of phenolic compounds and leaf extracts.

Pure test compounds quercetin (QUE), quercetin-3-*O*-rutinoside (RUT), chlorogenic acid (CGA), and caffeic acid (CAA) were applied in 1 mg mL^{-1} concentrations. Methanolic leaf extracts (0.3 mg leaf FW mL^{-1}) were from plants in one of the following treatment groups: untreated (C), H_2O_2 treated (H_2O_2), UV-B treated (UV-B), or treated with both UV-B and H_2O_2 (UV-B + H_2O_2).

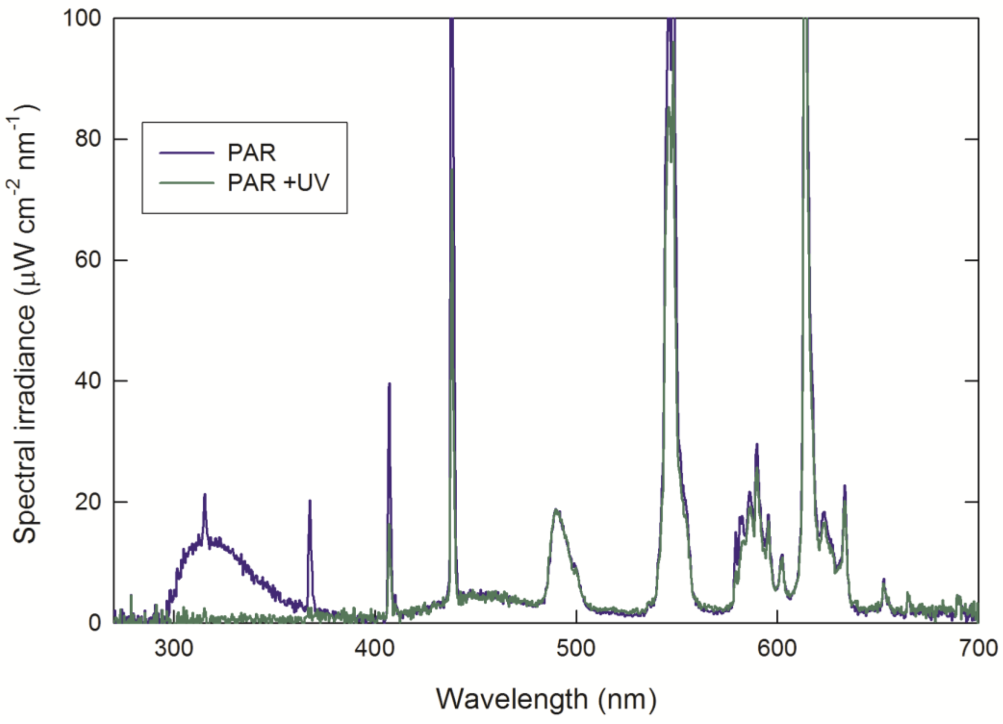

Supplementary Figure 3.

Spectral irradiation in the absence (green line) and in the presence (blue line) of supplemental UV radiation during the experiment.
